# Seismic Performance Evaluation of Corrosion-Damaged Reinforced Concrete Columns Controlled by Shear Based on Experiment and FEA

**DOI:** 10.3390/ma15186361

**Published:** 2022-09-13

**Authors:** Young-Shik Kim, Bok-Gi Lee, Ju-Seong Jung, Kang-Seok Lee

**Affiliations:** 1 Department of Architectural Engineering, Hanyang University, Ansan 15588, Korea; 2 Innovative Durable Building and Infrastructure Research Center, Hanyang University, Ansan 15588, Korea; 3 Department of Architectural Engineering & Smart City Engineering, Hanyang University, Ansan 15588, Korea

**Keywords:** reinforced concrete, shear column, corrosion, seismic performance, reduction factor, cyclic loading test, finite element analysis, half-cell potential

## Abstract

It is extremely important to investigate the effect of the seismic performance of corrosion-damaged reinforced concrete (RC) members, in terms of strength and deformability, on the seismic performance of the entire building. This will allow a more accurate assessment of the seismic performance of RC structures with corroded members, including beams and columns. However, current methods of evaluating the seismic performance of RC structures fail to fully consider the influence of reinforcement corrosion and other performance deterioration of RC members. The main objective of this study is to propose a practical method of evaluating the seismic performance of RC structures with corrosion-damaged members, identifying factors contributing to structural performance deterioration based on strength and deformability for direct, quantitative evaluation of seismic performance. To achieve the aforementioned objective, the authors examined the effects of reinforcement corrosion on the structural behavior of RC beams and factors contributing to structural performance deterioration. Past experiments verified the strong correlation between the half-cell potential (HCP) before and after reinforcement corrosion and the reduction factor based on energy absorption capacity. However, current research evaluates the correlation between the extent of corrosion and structural performance deterioration of RC beam members, which are not members that resist lateral force. As such, the results cannot be directly applied to the evaluation of the seismic performance of RC structures containing corrosion-damaged members. To achieve this study’s main purpose of proposing a practical method of evaluating the seismic performance of RC structures comprised of corrosion-damaged members, analytical methods including structural experiments should be applied to corrosion-damaged lateral resisting members, namely, column members of the shear failure type with non-seismic details. This study performed cyclic loading tests on columns of the shear failure type having reinforcement corrosion to examine the correlation between HCP before and after corrosion and seismic performance deterioration. At the same time, finite element analysis (FEA) was carried out in consideration of the weakened bonding between steel and concrete, so as to analyze the correlation between structural performance deterioration before and after corrosion of shear columns. Through a comparison of the experimental findings and FEA results, this study proposed a seismic performance reduction factor in relation to the extent of corrosion of shear columns.

## 1. Introduction

**Figure 1:**
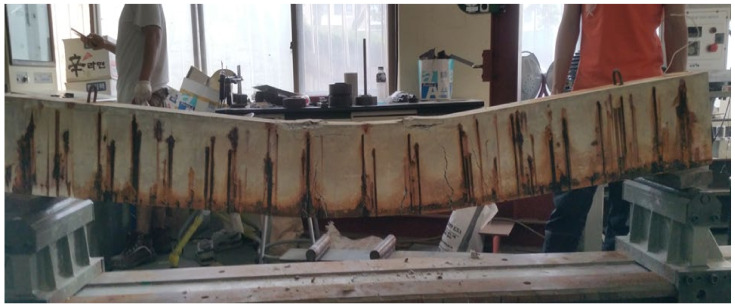
Structural performance deterioration of a reinforced concrete (RC) structure caused by reinforcement corrosion.

Reinforced concrete (RC) structures are one of the most common structural systems in modern society due to their low construction and maintenance costs and high durability compared to other types of structures. However, the structural performance of RC structures deteriorates over time for various reasons, such as defective construction and structural design errors, including changes in environmental conditions, design load, and material properties. Many RC structures are affected by reinforcement corrosion, one of the primary causes of deterioration [1,2,3,4,5,6,7,8,9,10,11]. The deterioration of structural performance leads to a shortened service life and reduced structural performance (see Figure 1).

The main sources of reinforcing rebar corrosion are chloride and carbonation, caused by atmospheric or environmental conditions, for instance, ocean, deicing salt, and pollution.

**Figure 2:**
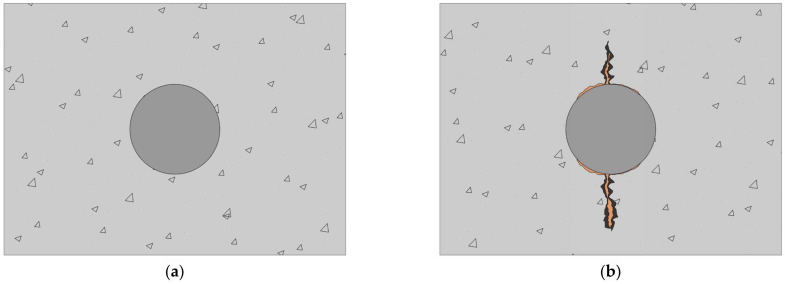

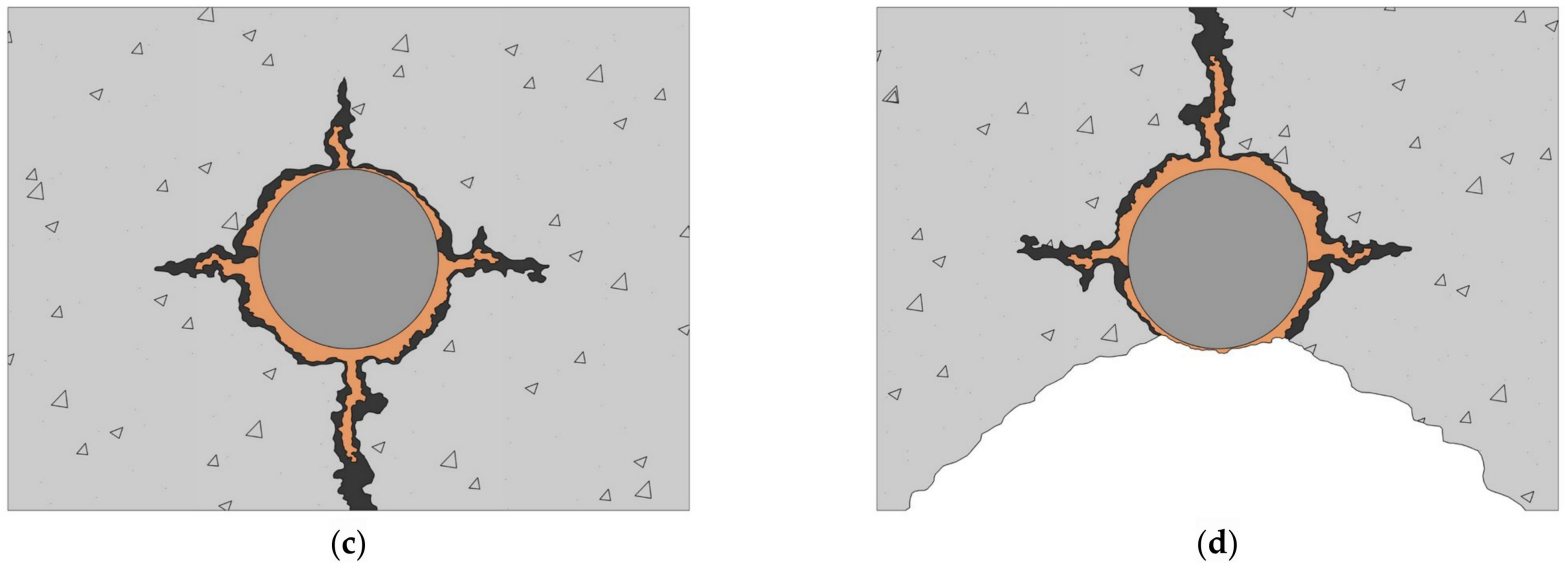
Corrosion mechanism of steel and effects of corrosion on RC members. (**a**) Before corrosion (**b**) Build-up of corrosion products. (**c**) Further corrosion and surface cracking. (**d**) Spalling of concrete.

The corrosion effects of RC columns and beams were vigorously examined by Hansson [2] and Shamsad [5], and the deterioration of members was investigated as follows (see Figure 2):

(1) Formation of a white patch: Calcium carbonate is generated by the reaction of carbon dioxide in the atmosphere in cement paste with calcium hydroxide. Calcium carbonate is precipitated on the concrete surface by moisture to form white spots;
(2) Brown pieces of steel: When corrosion starts, an iron oxide layer is formed on the upper part of the reinforcement, which is transported to the concrete surface by moisture;
(3) Crack formation: The corrosion product takes up more space than the original material, and the pressure applied to the concrete causes the crack. The larger the corrosion, the wider the crack;
(4) Concrete spalling: The bonding stress between concrete and reinforcement materials weakens, resulting in a decrease in rebar cross section;
(5) Steel rebar snap: Rebar snap occurs when the cross section is reduced;
(6) Bar buckling: The detachment or snaps of concrete on the reinforcement contributes to the buckling of the main reinforcement. Concrete bulges and affects the stability and life of RC buildings.

The decrease in yield strength arising from the reduced effective cross section of steel bars lowers the tensile force that the bars can withstand. In addition, the compressive strength is lowered by concrete spalling. The entire structure collapses due to the severe deterioration of structural performance.

**Figure 3:**
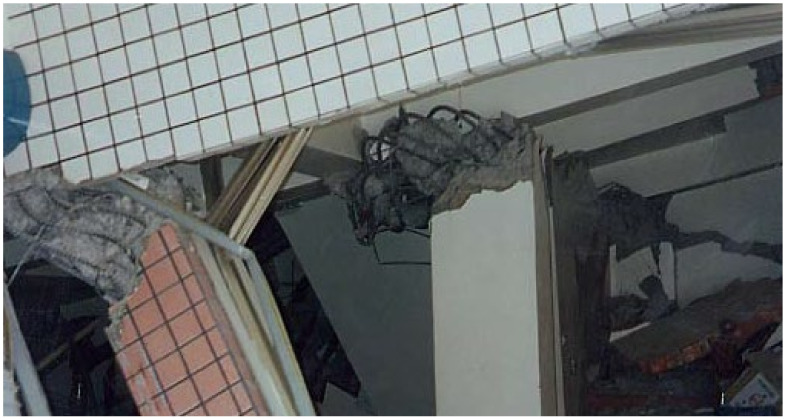
Corrosion of RC members damaged during the 1999 Jiji Earthquake in Taiwan.

The world has experienced frequent earthquakes in recent years. The severe corrosion of RC structures in earthquake-prone areas will inevitably have an impact on their seismic performance and safety. As shown in Figure 3, the reinforcement corrosion of RC members significantly affects the deterioration of structural performance caused by earthquakes, that is, seismic performance, as demonstrated by past earthquakes such as the 1995 Kobe Earthquake (Japan, M = 7.3), the 1999 Jiji Earthquake (Taiwan, M = 7.3), the 1999 Izmit Earthquake (Turkey, M = 7.4), the 2008 Sichuan Earthquake (China, M = 8.0), the 2010 Chile Earthquake (M = 8.8), the 2012 Great East Japan Earthquake (M = 9.0), the 2013 Lushan Earthquake (China, M = 7.0), and the 2016 Komamoto Earthquake (Japan, M = 7.0).

**Figure 4:**
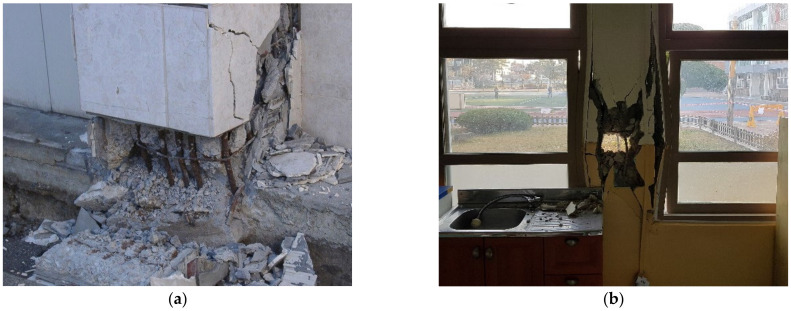
Corrosion of RC beams and columns damaged during earthquakes. (**a**) the Niigtaken Chuetsu Earthquake in Japan in 2004 and (**b**) the Pohang Earthquake in Korea in 2017.

As shown in Figure 4, the 2004 Niigtaken Chuetsu Earthquake (Japan, M = 6.8) and the 2017 Pohang Earthquake (Korea, M = 5.4) were of relatively low intensities, but the reinforcement corrosion of RC members caused damage to beams and columns [1]. Reinforcement corrosion not only reduces the cross-sectional area of steel but also weakens the bonds between steel and concrete over time. As a result, steel and concrete are no longer able to function as expected, and the lateral load resistance capacity drops significantly. As such, it is extremely important to investigate the effect of the seismic performance of corroded members, in terms of strength and deformability, on the seismic performance of the entire building. This will allow a more accurate assessment of the seismic performance of RC structures with corroded members, including beams and columns.

However, current methods of evaluating the seismic performance of RC structures, including FEMA (Federal Emergency Management Agency guidelines) 310 [12], FEMA 356 [13], and JBDPA (Japan Building Disaster Prevention Association standard) [14], fail to fully consider the influence of reinforcement corrosion and other performance deterioration of RC members.

According to the guidelines of FEMA 310 [12] and 356 [13], the seismic performance of RC structures must be evaluated in consideration of the extent of damage and impact on structural performance of each deteriorated member. As described above, the deterioration of concrete and steel significantly lowers the lateral load resistance capacity of RC members. Site inspections may be required to evaluate the effects of concrete and steel deterioration. The FEMA 310 guidelines do not provide procedures for quantitative analysis, limiting the evaluation of the seismic performance of RC structures to heuristic judgments.

Meanwhile, Japan’s standard for seismic evaluation [14] uses the formula I_S_ = E_O_ × S_D_ × T to evaluate the seismic performance of RC structures by story and direction. Here, E_O_ is the basic structural performance index calculated with respect to the ultimate lateral resistance, deformability, number of stories, and specific story concerned. S_D_ (irregularity index) is a subindex used to modify E_O_ according to irregularities in building shape and stiffness distribution relative to building height. T (aging index) is a subindex used to evaluate the time-dependent deterioration of a building.

The aging index (T) is used to evaluate the effects of structural deterioration or aging based on site inspections, which involve an initial inspection, a follow-up inspection, and an additional in-depth inspection. During the initial inspection, T = 0.8 is applied to buildings over 30 years old, while T = 0.9 is used in cases where internal finishing shows significant signs of spalling. The smallest T among T values obtained from site inspections is multiplied by Eo.

In the follow-up and additional in-depth inspection, the overall T is calculated by summing T_i_, which is the aging index of each story (i). For example, in the case of beams, T_i_ = 0.05 is adopted if more than 1/3 of the members inspected in each direction have reinforcement corrosion. T_i_ = 0.017 is adopted if reinforcement corrosion is between 1/9 to 1/3, T_i_ = 0.006 if below 1/9, and T_i_ = 0 if there are no beam members with reinforcement corrosion. In the case of columns, T_i_ = 0.15 is adopted if more than 1/3 of members inspected in each direction have reinforcement corrosion, T_i_ = 0.05 if between 1/9 and 1/3, T_i_ = 0.017 if below 1/9, and T_i_ = 0 if there is no reinforcement corrosion.

Unlike the guidelines of FEMA 310 and 356, Japan’s standard for seismic evaluation uses T for quantitative analysis of the seismic performance of RC structures with corroded members. However, this approach is not a direct method of evaluation that relies on structural performance reduction factor while taking into account the strength-ductility relationship between E_O_ and T. Instead, it indirectly evaluates the effects of reinforcement corrosion on overall seismic performance. As mentioned above, existing seismic performance guidelines evaluate the strength and deformability of the building structure itself using structural analysis software, which is focused on structural drawings and material strength measurements. In this case, the method of performance evaluation of corrosion-damaged RC members is indirect and qualitative, rather than direct and quantitative.

Most studies on seismic performance evaluation do not consider the effects of deterioration of RC members [8,15]. Moreover, the quantitative reduction factor, which looks at the strength-deformability correlation of corrosion-damaged members relative to intact members, has yet to be utilized. According to the necessity of quantitative performance reduction of reinforced concrete buildings due to corrosion, some researchers [16,17,18,19,20,21,22,23,24] have shown that reinforcement corrosion is the main cause of RC member deterioration and that it negatively affects bonding. J. G. Cabrera [16] and Ballim. Y et al. [23] reported that the bonding performance acting on the interface between concrete and reinforcement caused by the corrosion degree of the reinforcing bar was reduced, and the deformation occurring in the limit state of the beam member increased due to corrosion. R. Capozucca [17] and Bhargava, K et al. [19] presented a theory on the load-bearing capacity of reinforced concrete members reduced by corrosion. Jung, W.Y et al. [18] and Yang, X et al. [22] analyzed the performance degradation of RC members reduced by rebar corrosion through FEA. Table 1 summarizes the major research references related to this study among the previously conducted studies.

The seismic performance of RC structures containing corrosion-damaged members should be directly and quantitatively evaluated using the structural performance reduction factor based on strength-deformability, that is, energy dissipation capacity. This approach will allow more accurate evaluation of the seismic performance of RC structures with corrosion-damaged members, including beams and columns. The main objective of this study is to propose a practical method of evaluating the seismic performance of RC structures with corrosion-damaged members, identifying factors contributing to structural performance deterioration based on strength and deformability for direct, quantitative evaluation of seismic performance.

To achieve the aforementioned objective, the authors examined the effects of reinforcement corrosion on the structural behavior of RC beams and factors contributing to structural performance deterioration and published the results in reference [1]. The reference examined the effects of reinforcement corrosion on the structural behavior of RC beams and the structural performance reduction factor based on the strength-deformability correlation. For the experiment, eight shear beams and eight flexural beams were designed to evaluate the effects of reinforcement corrosion on shear and flexural failure, respectively. The impressed current technique was used to accelerate reinforcement corrosion.

The corrosion potential of reinforcing bars was quantitatively measured based on the half-cell potential (HCP), and a strong correlation was found between the structural performance reduction factor of corrosion-damaged beams and the volt-based average potential difference. That is, the correlation coefficient (R^2^) of flexural and shear beams was R^2^ = 0.78 and R^2^ = 0.91, respectively. The potential difference measured using the half-cell (HC) method can be used as an indicator of relative deterioration of structural performance if environmental conditions are constant. Additionally, also revealed was the possibility of evaluating energy absorption capacity (structural performance reduction factor) based on the strength-deformability of corrosion-damaged RC members, as well as a correlation between the reduction factor and average potential difference.

However, current research evaluates the correlation between the extent of corrosion and structural performance deterioration of RC beam members, which are not members that resist lateral force. As such, the results cannot be directly applied to the evaluation of seismic performance of RC structures containing corrosion-damaged members. To achieve this study’s main purpose of proposing a practical method of evaluating the seismic performance of the RC structures comprised of corrosion-damaged members, analytical methods including structural experiments should be applied to corrosion-damaged lateral resisting members, namely, column members of the shear failure type with non-seismic details.

**Figure 5:**
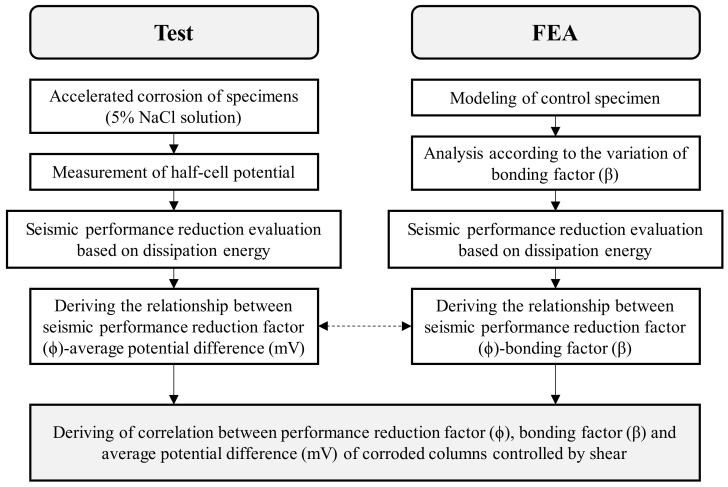
Flowchart of the research performed in this study.

As shown in Figure 5, this study performed cyclic loading tests on columns of the shear failure type having reinforcement corrosion to examine the correlation between HCP before and after corrosion and seismic performance deterioration. At the same time, FEA was carried out in consideration of the weakened bonding between steel and concrete, in order to analyze the correlation between structural performance deterioration before and after corrosion of shear columns. Through a comparison of the experimental findings and FEA results, this study proposed a seismic performance reduction factor in relation to the extent of corrosion of shear columns.

## 2. Experimental Program

### 2.1. Materials

The concrete column specimens had a compressive strength of 21 MPa, and cylindrical specimens were prepared using a cylindrical mold measuring 100 mm in radius and 200 mm in height according to ASTM C39/C39M [25]. The standard specimen calibration was 97% of the measured compressive strength, and the average compressive strength at 28 days was 21.4 MPa. D19 was used as longitudinal reinforcement and D10 as shear reinforcement. To determine the material properties of reinforcing bars, three tensile specimens each were prepared for the two types according to ASTM E8/E8M [26], and a tensile test was performed at 5 mm/min using a universal testing machine (UTM). The yield strength and tensile strength averaged 460 MPa and 495 MPa, respectively.

### 2.2. Design and Manufacture of Column Specimens

**Figure 6:**
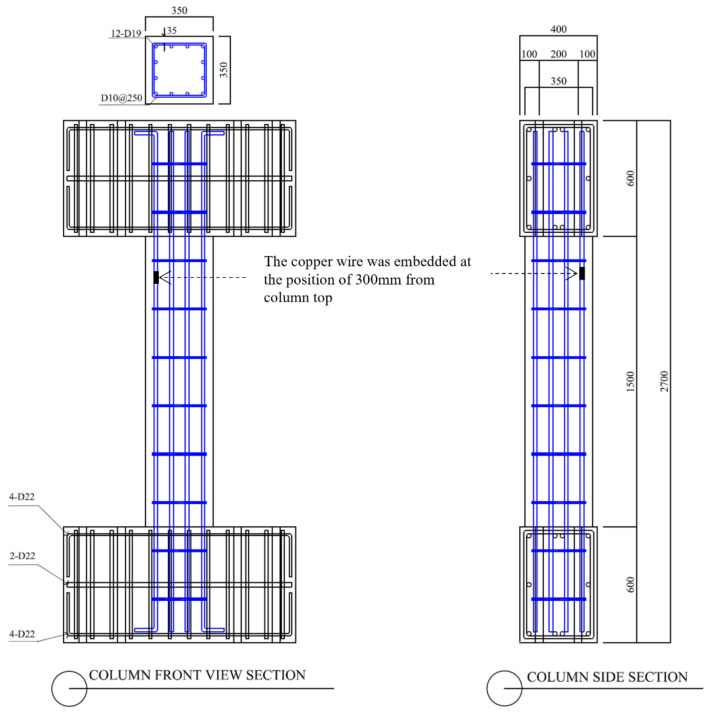
Reinforcement details of a column controlled by shear.

To investigate the effects of corrosion on the shear behavior of RC column members, five column specimens of the shear failure type were designed according to ACI 318 Building Code [27] (SC-C0 to SC-C4). As shown in Figure 6, the specimen has a square cross-section measuring 350 × 350 mm in width (b) and height (h). The bar arrangement and distance between shear reinforcements were adjusted to induce shear failure. 12-D19 was used for the main bars, and D10@250 for reinforcements. The clear span h was 1500 m, and the aspect ratio (h/D) was 4.3. Stubs were installed at the top of columns to take into account the confinement effect and were designed to be rigid and stiff to prevent influencing the behavior of the columns. Reinforcing bars were used to prevent cracks and local deformation when subjected to loads during experiments.

The SC-C0 specimen is a corrosion-free shear column that serves as a control, while the SC-C1, SC-C2, SC-C3, and SC-C4 specimens are corrosion-damaged specimens obtained by the impressed current technique. As shown in Figure 6, a copper wire was embedded at a height of 300 mm from the top of the column to accelerate corrosion.

**Figure 7:**
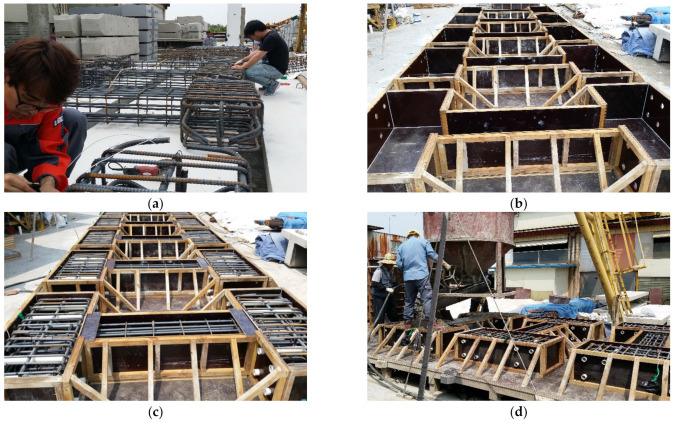
Column fabrication procedure: (**a**) assembly of reinforcement, (**b**) form production, (**c**) installation of wires for corrosion of reinforcement into form, and (**d**) placing of concrete.

Figure 7 shows the manufacturing process of the shear failure-type column specimens. After assembling reinforcing bar cages, copper wires were embedded to accelerate corrosion. Next, the assembled cages were placed within the formwork, and concrete was placed in the specimens.

### 2.3. Accelerated Corrosion of Reinforcement

**Figure 8:**
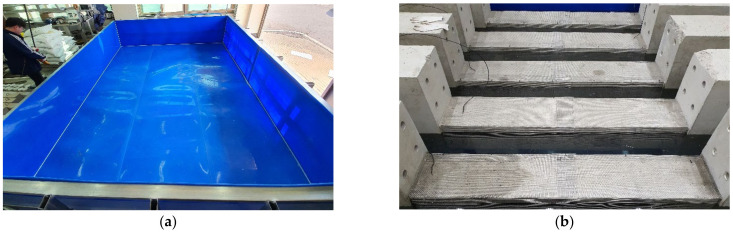

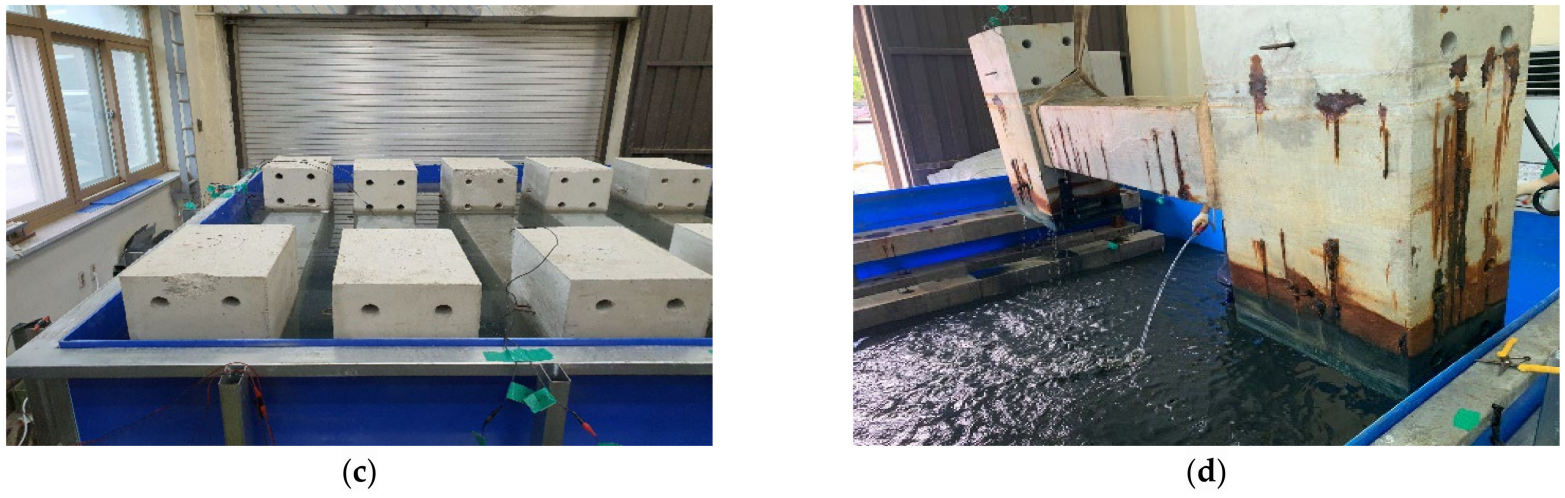
Accelerated corrosion setup: (**a**) tank with 5% NaCl solution, (**b**) immersion of column, (**c**) accelerated corrosion, and (**d**) corroded column.

The impressed current technique was used to accelerate reinforcement corrosion. As shown in Figure 8, the column specimens were arranged horizontally in a tank 30 days after casting with 5% NaCl solution as an electrolyte. The solution level in the tank was adjusted to approximately 2 cm below the top surface of the horizontal specimens to ensure that all reinforcement was properly sunk. Direct current was then supplied to the specimens. Reinforcing bars with embedded copper wires were used as the anode and external stainless-steel plates as the cathode. To simulate the deterioration of seismic performance in relation to reinforcement corrosion, it is important to set appropriate durations for the accelerated corrosion process. The duration of accelerated corrosion increased with specimen number from C1 to C4, and structural deterioration also increased accordingly.

The specimens, SC-C1, SC-C2, SC-C3, and SC-C4, were removed in order from the tank and dried. The potential field of the concrete surface was then measured using a half-cell electrode and a high-impedance voltmeter, which are among the recommended devices for measurement of corrosion potential in ASTM C876 [28]. The measurement area covered the column width of 350 mm and a height of 450 mm from the top of the column. This area, measuring 350 × 450 mm was divided into a 5 × 5 mm grid. HCP was measured at a total of 63 points, with 7 points in each row and 9 points in each column with reference to the center of the grid.

### 2.4. Test Program

**Figure 9:**
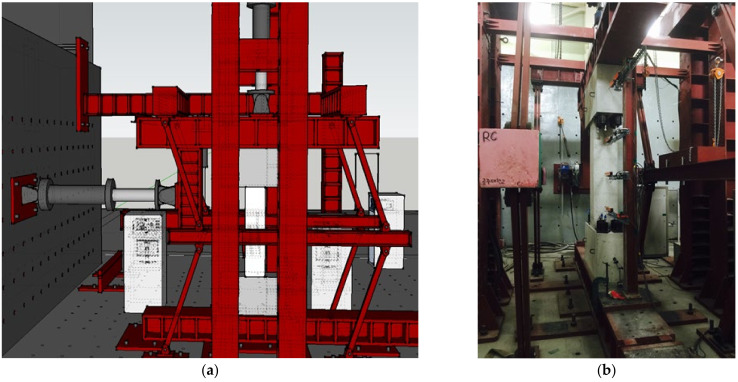
Experimental configuration for the cyclic loading tests: (**a**) 3D-diagram and (**b**) photograph.

Figure 9 shows the experimental configuration for cycling loading tests on corrosion-damaged shear failure-type specimens. The method employed in this study was developed by the Building Research Institute [29] and is widely used in the evaluation of the seismic behavior of columns. In the experiment shown in Figure 9, the line of action of lateral load passes through the center of a column specimen via the L-shaped steel frame installed at the top, and lateral shear force is effectively applied.

The axial force of 0.1 f_ck_A_g_, or 257.3 kN, was constantly applied using the 1000 kN actuator installed at the top, and lateral load was applied under the displacement control method using the 1000 kN actuator installed at the reaction wall. The loading point of the actuator, which applies a lateral force to achieve an asymmetric moment, was designed to coincide with the center of the specimen. As shown in Table 2, each loading step had three loading cycles, and lateral drift increased with drift angle (R), which was varied from 0.1%, 0.2%, 0.3%, 0.4%, 0.5%, 0.67%, 0.83%, 1%, 1.17%, 1.33%, 1.67%, to 2%. Lateral d was measured using five linear variable differential transducers (LVDTs), one each at the top and bottom stubs, and three at different sections of the column.

## 3. Test Results and Discussion

### 3.1. Half-Cell Potential of Reinforced Concrete Columns

**Figure 10:**
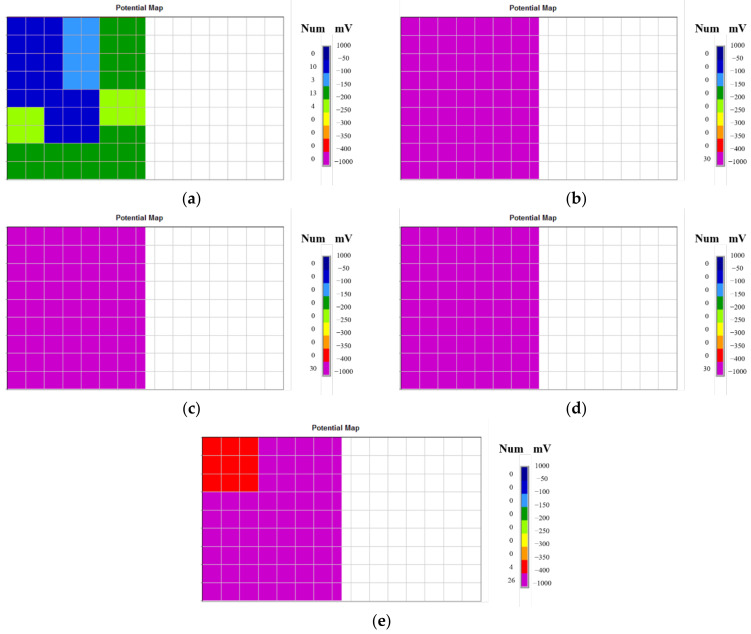
Potential maps of shear-controlled columns: (**a**) SC-C0 (−125 mV), (**b**) SC-C1 (−405 mV), (**c**) SC-C2 (−545 mV), (**d**), SC-C3 (−575 mV), and (**e**) SC-C4 (−605 mV).

This study used the CANIN+ Corrosion Analyzing Instrument [30], developed by Proceq, to measure the corrosion potential of reinforcing bars. As specified in ASTM C876 [28], the temperature was set to approximately 28 °C when measuring the corrosion potential of reinforcing bars embedded in concrete. Table 3 shows the average voltage (mV CSE) measured at 63 points for the reinforcement corrosion potential of each column specimen. The potential difference is increasing in the order of specimen number from SC-C0 to SC-C4, which can be traced to the change in the rate of accelerated corrosion of corrosion-damaged specimens under the impressed current technique. Figure 10 shows the HCP map for each of the 63 measurement points.

According to ASTM C876 [28], there is a greater than 90% possibility of an area not having reinforcement corrosion if the measured potential is higher than −200 mV CSE (Copper Sulfate Electrode). If the measured potential is a larger negative than −350 mV CSE, the possibility of reinforcement corrosion occurring in that area is over 90%. The HCP of the SC-C0 column specimen, which did not accelerate the corrosion process, was −125 mV CSE, or higher than −200 mV CSE. The values for other specimens (SC-C1, SC-C2, SC-C3, and SC-C4), which contributed to accelerated corrosion, were more negative than −350 mV CSE.

### 3.2. Cracking and Failure Pattern

(1) SC-C0 (Control Specimen)

**Figure 11:**
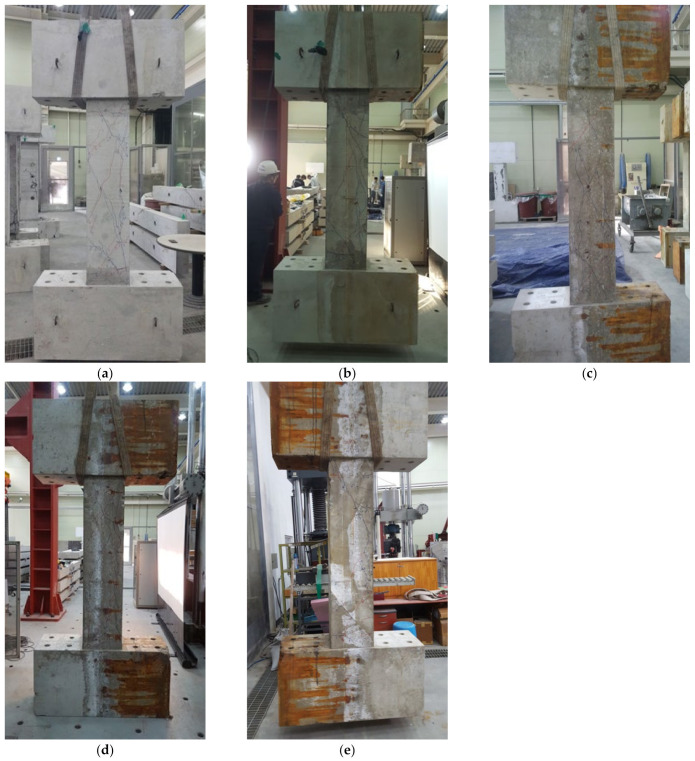
Cracking and failure patterns of columns controlled by shear: (**a**) SC-C0, (**b**) SC-C1, (**c**) SC-C2, (**d**), SC-C3, and (**e**) SC-C4.

Figure 11a shows the final failure of the SC-C0 control specimen. This specimen was designed to obtain a reference value of energy dissipation capacity and to examine how seismic performance deterioration is related to corrosion by comparing cycling loading results to those of corrosion-damaged specimens.

SC-C0 developed initial flexural cracks at the bottom with positive loading at the sixth cycle (R = 0.2%) but not at the midspan. Later, shear cracks were observed at the top and bottom, and at the same time, they gradually expanded to the midspan. Many shear cracks developed at the top, bottom, and midspan at the 12th cycle (R = 0.4%) of positive loading. The width of cracks gradually increased at the 18th cycle (R = 0.67%) and rapidly widened when the top and bottom shear cracks joined each other at the 24th cycle (R = 1%), with many cracks measuring more than 1 mm in width. At the 30th cycle (R = 1.33%), during which the lateral displacement was 20 mm, the broadened width of shear cracks at the midspan caused concrete spalling, and eventually resulted in shear failure.

(2) SC-C1 to SC-C4 (Corroded Specimens)

Figure 11b–e shows the final failure patterns of SC-C1 to SC-C4 specimens, which are column specimens that were subject to corrosion acceleration. They were fabricated for the purpose of examining the deterioration in seismic performance in relation to the extent of corrosion.

The specimens from SC-C1 to SC-C4 exhibited similar structural behavior to SC-C0. Small flexural cracks with a width of 0.1 mm were seen at the bottom of columns at the sixth cycle (R = 0.2%) of positive loading, but not at the midspan. Later, more shear cracks developed at the top and bottom and gradually expanded to the midspan. At the 12th cycle (R = 0.4%) of positive loading, multiple shear cracks formed at the top, bottom, and midspan. At the 15th cycle (R = 0.5%), the shear cracks widened rapidly compared to the control specimen. At the 21st cycle (R = 0.83%), the shear cracks at the top joined those at the bottom, and many shear cracks larger than 1 mm developed. At the 25th cycle (R = 1.17%), during which the lateral displacement was 17.5 mm, shear cracks measuring 3–3.5 mm formed at the midspan, leading to concrete spalling and shear failure.

### 3.3. Load-Displacement and Seismic Performance Reduction Factor

**Figure 12:**
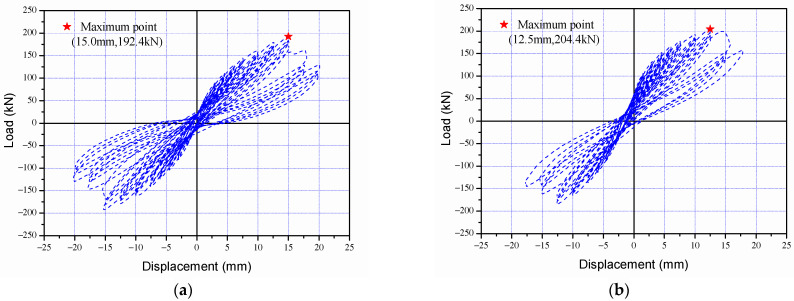

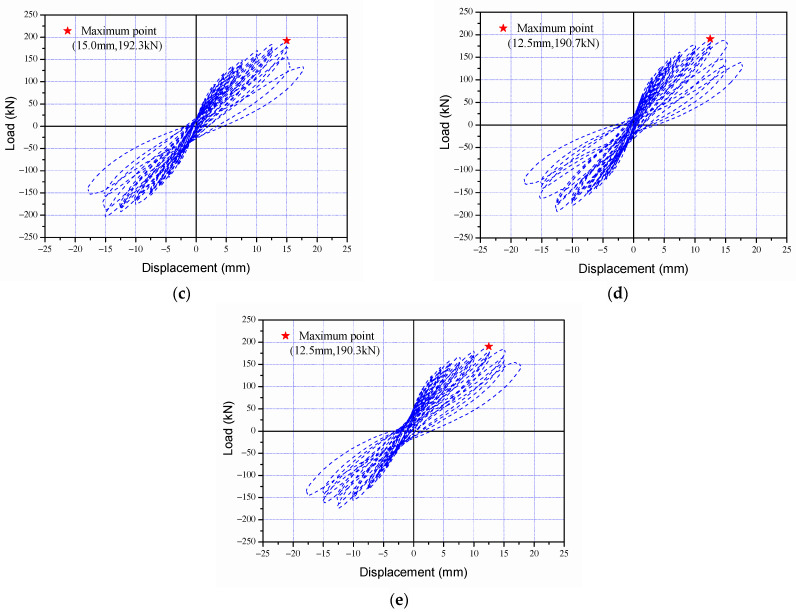
Load and displacement curves of column specimens following the cyclic loading tests: (**a**) SC-C0 specimen, (**b**) SC-C1 specimen, (**c**) SC-C2 specimen, (**d**) SC-C3 specimen, and (**e**) SC-C4 specimen.

**Figure 13:**
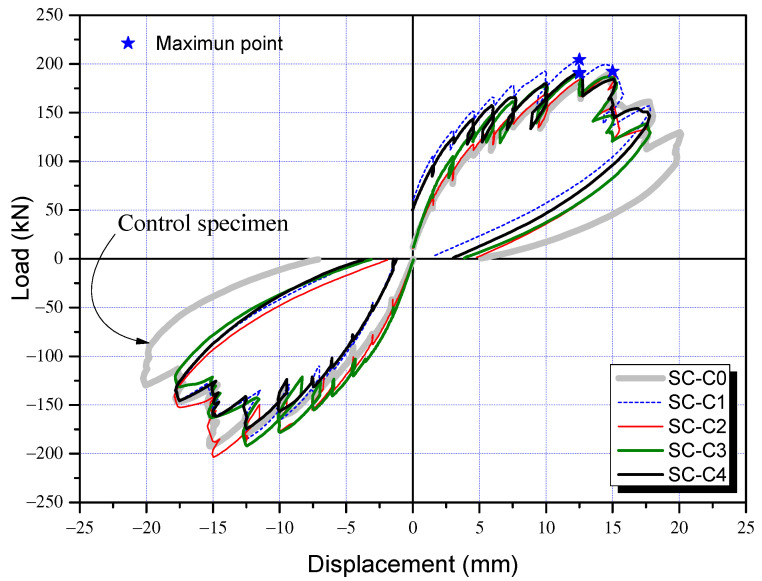
Envelope of the load-displacement relations of the specimens.

**Figure 14:**
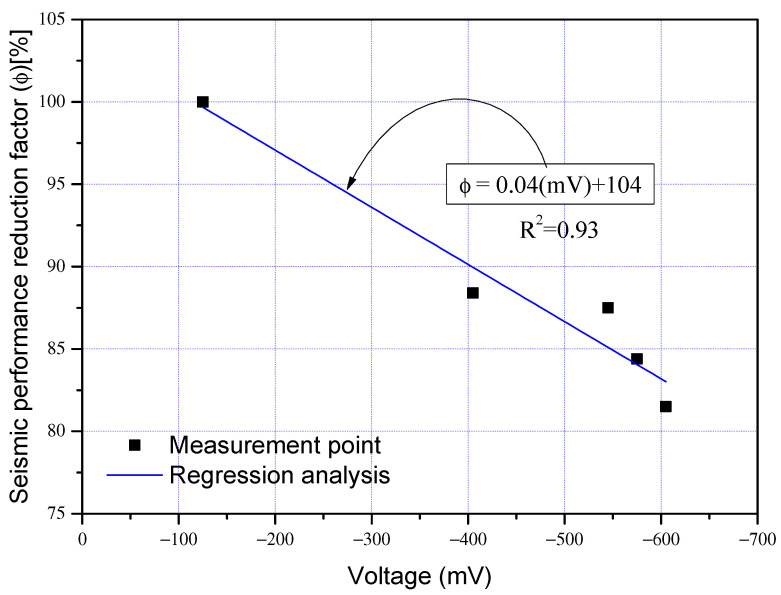
Relationship between the seismic performance reduction factor and average potential difference in terms of voltage.

The load-displacement curves of the control specimen (SC-C0) and corrosion-damaged specimens (SC-C1 to SC-C4) are presented in Figure 12, and the envelope relations of load-displacement curves in Figure 13. As shown in Figure 13, no significant decrease was observed in the extreme load and extreme displacement of the corrosion damage specimens (SC-C1 to SC-C4) compared to the control specimen (SC-C0). Therefore, energy dissipation, which may include deformation capacity and load capacity as indicators of performance degradation, was additionally analyzed. Table 4 gives the ultimate load and ultimate displacement of each shear column, as well as the energy dissipation area calculated based on strength and deformation, and the seismic performance reduction factor (ϕ) [%] derived using Equation (1). Figure 14 shows the correlation between the average potential difference in terms of voltage (mV) and seismic performance reduction factor (ϕ), as previously outlined in Table 4.

(1) $$\varphi =\frac{E_{r}}{E_{t}}\times 100$$

Here, *E_t_* and *E_r_* are the energy dissipation areas of column members before and after reinforcement corrosion.

According to Figure 12 and Figure 13 and Table 4, the SC-C0 control specimen had an ultimate load of 192.4 kN, an ultimate displacement of 15.0 mm, and a dissipation energy of 2106.7 kN·mm. The results for the corrosion-damaged SC-C1, measured at an average potential difference of −405 mV, show an ultimate load of 204.4 kN, an ultimate displacement of 12.5 mm, and dissipation energy of 1861.8 kN·mm. For the SC-C2 specimen (−545 mV), the values were calculated as 192.3 kN, 15.0 mm, and 1843.8 kN·mm, respectively. The SC-C3 (−575 mV) and SC-C4 (−605 mV) specimens had smaller average potential differences. Their ultimate loads were 190.7 kN and 190.3 kN, respectively. Displacement was both 12.5 mm and dissipation energy was 1778.1 kN·mm and 1718.9 kN·mm, respectively.

The dissipation energy of each specimen presented in Table 4 is closely related to the seismic performance reduction factor defined in Equation (1) and indicates that the reduction factor is decreasing in relation to the extent of corrosion (HCP). As shown in Figure 14, a strong correlation of R^2^ = 0.93 was found between the seismic performance reduction factor and the average potential difference of corrosion-damaged column members. Equation (2), based on regression analysis, can be used to obtain approximate values of mV and ϕ of shear failure-type column specimens.

(2) $$\phi =0.04\left(\mathrm{mV}\right)+104\;\left(Test\;:\;R^{2}=0.93\right)\;$$

## 4. FEA Model of Shear Failure-Type Columns and Their Results

Based on past results [18,19,20,21,22,23,24] demonstrating the strong correlation between reinforcement corrosion and bonding performance reduction, this study performed FEA on the effects of weakened bonds on seismic performance deterioration based on strength-deformability of column specimens and determined the correlation between bonding factor (β) and seismic performance reduction factor (ϕ). By comparing β–ϕ obtained from FEA and the mV–ϕ relationship previously depicted in Figure 14, β and mV were integrated from the perspective of ϕ and expanded to establish the ϕ–β–mV interaction diagram.

### 4.1. Overview of FEA

#### 4.1.1. Overview of Analysis Software

The analysis model used in FEA was Vecchio’s disturbed stress field model (DSFM) [31], an extension of the modified compression field theory (MCFT) [32]. Similar to MCFT, DSFM is a rotating-angle model that assumes that the direction of cracks matches the direction of compressive stress when inducing adequate conditions for force equilibrium and strain. However, DSFM takes into account the deformation caused by shear slip on cracked surfaces in addition to average deformation. Like the truss model, it considers force equilibrium conditions and adequate conditions of strain, thereby enabling the prediction of not only flexural strength and flexural deformation, but also shear strength and shear deformation. DSFM has been incorporated into the two-dimensional non-linear FEA software VecTor2 [33], which is used to predict nonlinear responses of RC structures.

Using VecTor2 [33], this study examined the effects of weakened bonding on strength-deformability of shear failure-type column specimens, presented in Figure 6, that is, the correlation between bonding factor (β) and structural performance reduction factor (ϕ).

Table 5 summarizes the constitutive and behavioral models used for concrete elements, reinforcement elements, and bond elements in this research, and the convergence limit was set to 1.00001 for the reliability of the analysis.

#### 4.1.2. Concrete Analysis Model

(1) Concrete Stress-Strain Model

This study employed the parabola model proposed by Hognestad [34] in Equation (3), which is suitable for modeling compression pre-peak response for normal strength concrete below 40 MPa.

(3) $$f_{ci}=-f_{p}\left\{2\left(\frac{\varepsilon_{ci}}{\varepsilon_{p}}\right)-{\left(\frac{\varepsilon_{ci}}{\varepsilon_{p}}\right)}^{2}\right\}<0\;\mathrm{for}\;\varepsilon_{ci}<0$$

Here, $f_{ci}\;$= principal compressive stress, $f_{p}\;$= peak compressive stress, $\varepsilon_{ci}$ = principal compressive strain, and $\varepsilon_{p}$ = peak compressive strain.

**Figure 15:**
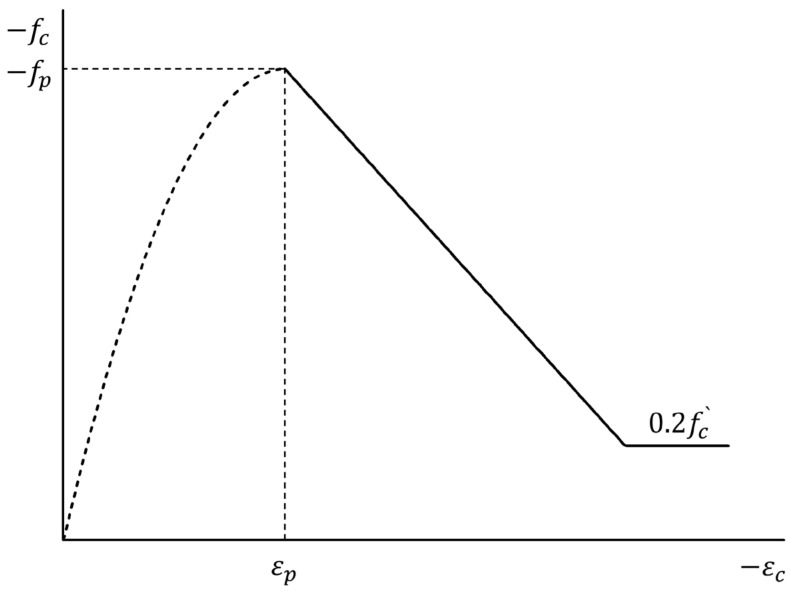
Modified Park-Kent model for post-peak concrete compression response.

The model for compression post-peak response developed by Kent and Park [35] was modified by Park, Priestly, and Gill [36]. This study applied the modified Park-Kent model, as presented in Equation (3) and Figure 15.

(4) $$f_{ci}^{b}=-\left[f_{p}+Z_{m}f_{p}\left(\varepsilon_{ci}-\varepsilon_{p}\right)\right]<0\;or-0.2\;f_{p}\;\mathrm{for}\;\varepsilon_{ci}<\varepsilon_{p}<0$$

Here, $f_{ci}^{b}=func\left(\varepsilon_{ci}\right)$, $Z_{m}=\frac{0.5}{\frac{3+0.29\left|f_{c}^{\prime }\right|}{145\left|f_{c}^{\prime }\right|-1000}\cdot \left(\frac{\varepsilon_{0}}{-0.002}\right)+{\left(\frac{\left|f_{lat}\right|}{170}\right)}^{0.9}+\varepsilon_{p}}$, $f_{c}^{\prime }$ = unconfined uniaxial concrete cylinder strength, $\varepsilon_{0}$ = strain responding to $f_{c}^{\prime }$, and $f_{lat}=f_{c1}+f_{c2}+f_{c3}-f_{ci}\leq 0$ (i = 1 or 2), the summation of principal acting transversely to the direction under consideration.

(2) Concrete Compression Softening Model

**Figure 16:**
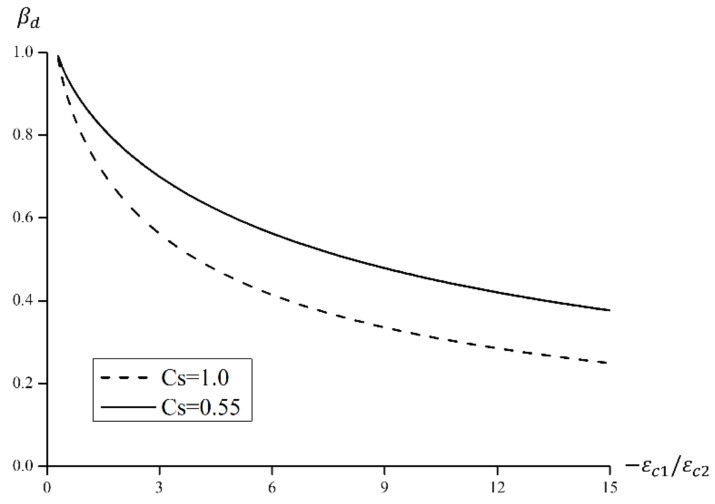
A compression softening model.

The compressive behavior of concrete in the main tensile direction under biaxial stress is different from stress-strain behavior in the main compressive direction under uniaxial stress due to the influence of cracks. As main tensile stress increases, the compressive stress decreases relative to behavior exhibited under uniaxial stress, and this is known as the compression softening effect [32]. This study took into account the compression softening effect by using the Vecchio 1992-A [31] model, presented in Equation (5) and Figure 16.

(5) $$f_{p}=\beta_{d}f_{c}^{\prime },\;\varepsilon_{p}=\beta_{d}\varepsilon_{c}$$

Here, $\beta_{d}=func\left(\frac{\varepsilon_{c1}}{\varepsilon_{0}}\right)=\frac{1}{1+C_{s}C_{d}}\leq 1$, $C_{d}=\left\{\begin{array}{c} 0\\ 0.35{\left(r-0.28\right)}^{0.80} \end{array}\right.$ $\begin{array}{c} \mathrm{if}\;r<0.28\\ \mathrm{if}\;r>0.28 \end{array}$, $r=\frac{-\varepsilon_{c1}}{\varepsilon_{c2}}\leq 400$, $C_{s}=\left\{\begin{array}{c} 0\\ 0.55 \end{array}\right.\begin{array}{c} \;\mathrm{if}\;\mathrm{shear}\;\mathrm{slip}\;\mathrm{not}\;\mathrm{considered}\\ \mathrm{if}\;\mathrm{shear}\;\mathrm{slip}\;\mathrm{considered} \end{array}$.

The compression softening model shown in Figure 16 was developed from experiments on 116 panels and shell elements. It limits the ratio of compressive strain-stress to 400 to prevent overestimation of the softening effect when reinforcing bars yield under large tensile strain. The coefficient Cs indicates whether or not shear slip is considered.

(3) Concrete Tension Stiffening Model

**Figure 17:**
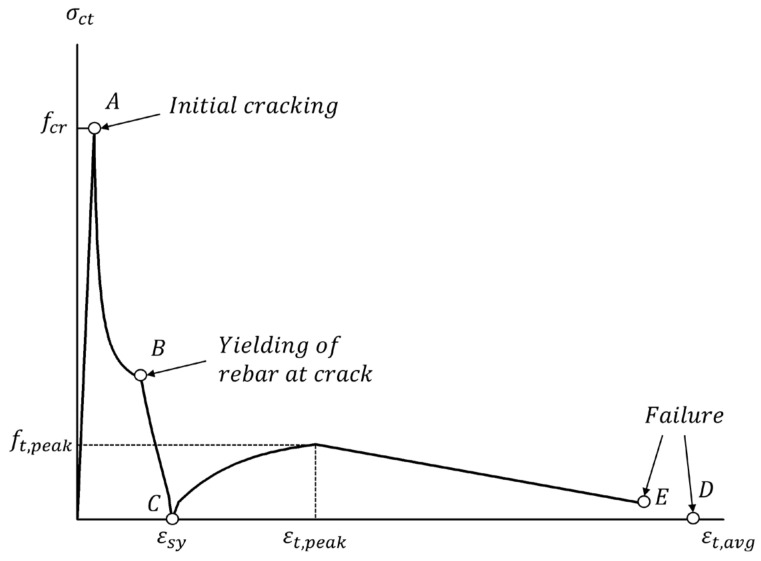
A tension stiffening model.

Stiffness decreases when cracks develop in RC structures, and stress is redistributed on cracked surfaces. Reinforcing bars bear all tensile forces at cracked cross-sections, but as cracks continue to develop with increasing loads, the concrete between cracked surfaces begins to bear some of the tensile forces. The concrete causes an increase in the tensile stiffness of reinforcing bars, which is known as the tension stiffening effect [37]. If the tensile strength of concrete is neglected, the concrete tensile stress immediately drops to 0 upon cracking and becomes redistributed across all reinforcing bars. This is reflected in the discontinuous changes in stiffness, which can result in unrealistic variations in load-displacement response. To take into account the tension stiffening effect, this study used the model of Lee et al. [37] shown in Equation (6) and Figure 17 and determined average deformation conditions by calculating tensile stress at cracking positions in concrete.

(6) $$f_{ct,avg}=\left\{\begin{array}{c} f_{ct,peak}-f_{ct,peak}{\left(\frac{\varepsilon_{t,peak}-\varepsilon_{t,avg}}{\varepsilon_{t,peak}-\varepsilon_{sy}}\right)}^{2}\;for\;\varepsilon_{sy}\leq \varepsilon_{t,avg}\leq \varepsilon_{t,peak}\\ f_{ct,peak}-\frac{f_{ct,peak}{}^{-0.5f_{ct,peak,\rho_{min}}}}{0.1-\varepsilon_{t,peak}}\left(\varepsilon_{t,avg}-\varepsilon_{t,peak}\right)\geq 0.5\;f_{ct,peak,\rho_{min}}\;for\;\varepsilon_{t,avg}\geq \varepsilon_{t,peak} \end{array}\right.$$

Here, $f_{ct,avg}$ = average tensile stress of concrete, $f_{ct,peak}$ = peak average tensile stress of concrete after reinforcement yielding ($f_{ct,peak}=a\sqrt{f_{c}^{’}}$), $a=-0.0313p_{s}^{0.57}d_{b}+3.3881p_{s}^{0.76}$, $\varepsilon_{t,peak}$ = peak tensile strain of reinforced concrete ($\varepsilon_{t,peak}=0.01+0.001\cdot \max\left(15-d_{b},0\right)\geq \varepsilon_{sh}$), $\varepsilon_{t,avg}$ = average tensile strain of reinforced concrete, $\varepsilon_{sy}$ = yield strain, $f_{ct,peak,\rho_{min}}$ = $f_{ct,peak}$ with consideration of $\rho_{min}$, $\rho_{min}=\frac{\varepsilon_{cr}\cdot E_{c}}{f_{sy}-\varepsilon_{cr}\cdot E_{s}}$, $\varepsilon_{cr}$ = cracking strain of concrete, $f_{sy}$ = yield strength of steel reinforcement.

#### 4.1.3. Steel Reinforcement Analysis Model

For stress-strain modeling of steel reinforcement, this study used the curvilinear model in Equation (7), which considers strain hardening and necking after the perfect plasticity region. The modulus of elasticity was 200 GPa.

(7) $$f_{s}=f_{su}-\left(f_{su}-f_{sy}\right){\left(\frac{\epsilon_{su}-\epsilon_{s}}{\epsilon_{su}-\epsilon_{sh}}\right)}^{4}$$

Here, $f_{s}$ = tensile stress of steel reinforcement, $f_{su}$ = rupture strength of steel reinforcement, $f_{sy}$ = yield strength of steel reinforcement, $\epsilon_{su}$ = rupture strain of reinforcement, and $\epsilon_{sh}$ = hardening strain of reinforcement.

**Figure 18:**
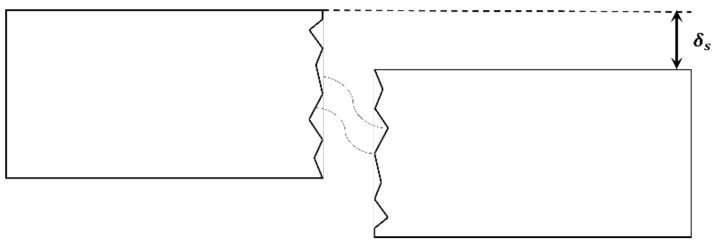
Dowel resistance mechanism.

As shown in Figure 18, the dowel action of steel reinforcement increases sheer resistance as cracks slip across longitudinal reinforcements. This dowel action plays an important role in determining the shear strength of RC members and their softening after strain hardening. In this study, the dowel action of steel reinforcement was modeled using the Tassios model [38], given in Equation (8).

(8) $$V_{d}=E_{s}I_{z}\lambda^{3}\delta_{s}\leq V_{du}$$

Here, $V_{d}$ = dowel force developed in the reinforcement bar, $E_{s}$ = modulus of elasticity of steel reinforcement bar, $I_{z}=\frac{\pi d_{b}^{4}}{64}$, $\lambda =\sqrt[{4}]{\frac{k_{c}d_{b}}{4E_{s}I_{z}}}$, $k_{c}=\frac{127\cdot c\sqrt{f_{c}^{\prime }}}{d_{b}^{\;2/3}}$, c=0.8, $\delta_{s}$ = shear slip along the crack, $d_{b}$ = diameter of the reinforcement, $V_{du}$ = dowel force developed in the reinforcement bar at ultimate limit state ($V_{du}=1.27d_{b}^{2}\sqrt{f_{c}^{\prime }f_{y}}$), $f_{c}^{\prime }$ = compressive strength of the concrete, and $f_{y}$ = yield strength of the reinforcement.

**Figure 19:**
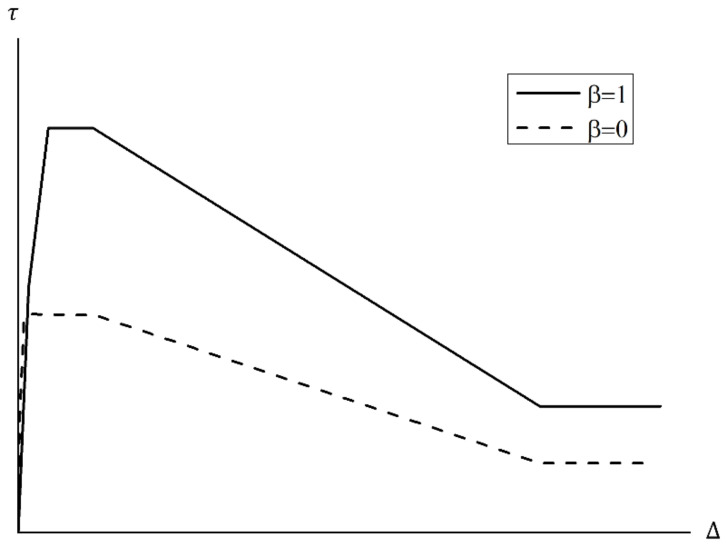
Eligehausen bond stress-slip response.

According to past studies [18,19,20,21,22,23,24], weakened bonding is highly related to the corrosion of reinforcements of RC members. This study used the model proposed by Eligehausen et al. [39], shown in Figure 19, as a steel reinforcement bonding model. Under this model, the bond stress-slip relationship is determined by: (1) an ascending non-linear branch; (2) a constant bond stress plateau; (3) a linearly declining branch; and (4) sustaining residual stress branch based on the bonding factor β given in Equation (9).

(9) $$\tau =\tau_{sp1}{\left(\frac{\Delta }{\Delta_{sp1}}\right)}^{a}\;for\;\Delta \leq \Delta_{sp1}\;\tau =\tau_{sp1}-\left[\frac{\left(\Delta -\Delta_{sp12}\right)}{\left(\Delta_{sp3}-\Delta_{sp2}\right)}\left(\Delta_{sp2}-\tau_{spf}\right)\right]\;for\;\Delta_{sp1}<\Delta \leq \Delta_{sp2}\;\tau =\tau_{sp2}-\left[\frac{\left(\Delta -\Delta_{sp1}\right)}{\left(\Delta_{sp3}-\Delta_{sp2}\right)}\left(\Delta_{sp2}-\tau_{spf}\right)\right]\;for\;\Delta_{sp2}\text{<}\Delta \leq \Delta_{sp3}\;\tau =\tau_{spf}\;for\;\Delta_{sp3}<\Delta$$

Here, τ = bond stress along the reinforcing bar, Δ = bond slip, $\tau_{sp1}$= bond stress at an ascending non-linear branch ($\tau_{sp1}=\tau_{s1}+\beta \left(\tau_{p1}-\tau_{s1}\right)$), $\Delta_{sp1}$ = bond slip at an ascending non-linear branch ($\Delta_{sp1}=\Delta_{s1+}\beta \left(\Delta_{p1}-\Delta_{s1}\right)\geq \Delta_{s1}$), $\tau_{sp2}$= bond stress at a constant bond stress plateau ($\tau_{sp2}=\tau_{sp1}$), $\Delta_{sp2}$ = bond slip at a constant bond stress plateau ($\Delta_{sp2}=\Delta_{p2}$), $\tau_{spf}$ = bond stress at a sustaining residual stress branch ($\tau_{spf}=\tau_{s1}+\beta \left(\tau_{pf}-\tau_{sf}\right)$), $\Delta_{sp3}$ = bond slip at a sustaining residual stress branch ($\Delta_{sp3}=\Delta_{p3}$), and β = confinement pressure bonding factor.

#### 4.1.4. FEA Model

**Figure 20:**
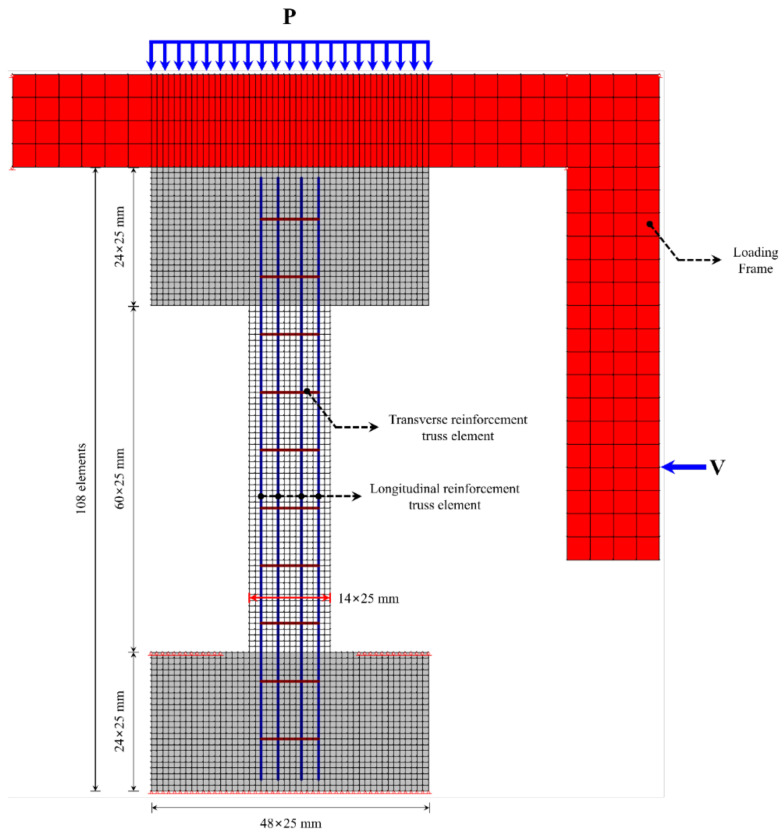
Finite element model of a column controlled by shear.

Based on the analysis models for concrete and steel reinforcement described in Section 4.1.2 and Section 4.1.3, this study performed finite element modeling of column specimens controlled by shear, designed according to the dimensions of Figure 6. Figure 20 shows a finite element model of a column controlled by shear performed in this study.

The top and bottom stubs of the column specimens had 48 × 24 elements over a width of 1200 mm and a height of 600 mm, amounting to a total of 1152 specimens. The columns themselves had 14 × 60 elements over a width of 350 mm and a height of 1500 mm, amounting to a total of 840 elements.

In FEA, β (confinement pressure bonding factor) was set as the main variable to determine the effects of weakened bonding resulting from reinforcement corrosion on strength-deformability, that is, the correlation between bonding factor (β) and seismic performance reduction factor. With β=1 as the perfect bonding state, the analysis was carried out with 13 variables with β up to 0.

### 4.2. FEA Results

**Figure 21:**
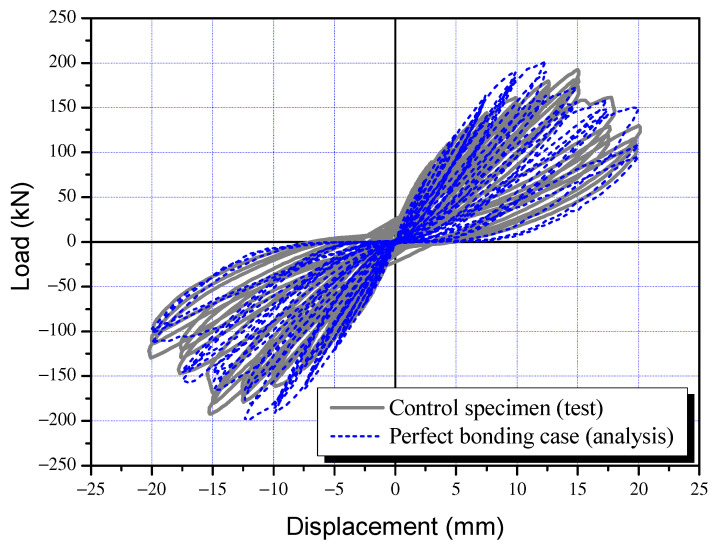
Comparison between test and analysis results of the control shear column specimen.

Figure 21 compares the experimental results (SC-C0) and FEA results (Case-1, β=1) of the control specimen. Table 5 presents the results of the analysis carried out using 13 variables with β=1 as a reference and up to 0.0. The ultimate load of the control specimen was found to be 202.9 kN (Table 6), which is about 105% of the experimental value of 192.4 kN (Table 3). The ultimate displacement at this point was 12.5 mm in the analysis, about 83% of the experimental value of 15.0 mm. The dissipation energy was found to be 2105.9 kN-m, which is highly similar to the experimental value of 2106.7 kN. The analysis results using VecTor2 were consistent with experimental observations. The proposed FEA model can be seen as effective in examining the effects of weakened bonding on structural performance reduction based on strength-deformability, that is, the correlation between bonding factor (β) and seismic performance reduction factor (ϕ).

**Figure 22:**
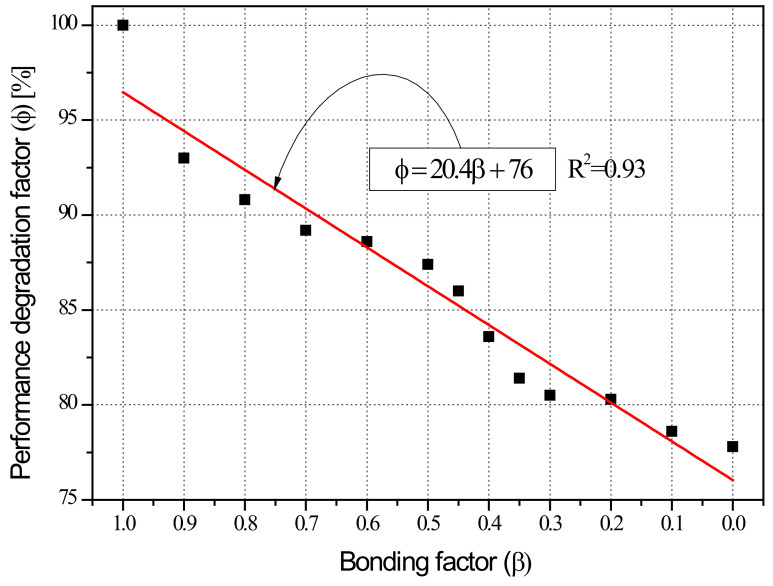
Relationship between bonding factor (β) and seismic performance reduction factor (ϕ) of shear column specimens (FEM analysis).

Figure 22 shows the correlation between bonding factor (β) and seismic performance reduction factor (ϕ) for each case presented in Table 6. The bonding factor (β) and the structural performance reduction factor (ϕ) can be approximated using Equation (10), obtained by regression analysis. The correlation coefficient of β-ϕ was R^2^ = 0.93, which indicates a very strong correlation. It was possible to approximate the mV and ϕ of column specimens used in experiments using Equation (2).

(10) $$\phi =20.4\beta +76\;\left(Analysis\;:R^{2}=0.93\right)$$

**Figure 23:**
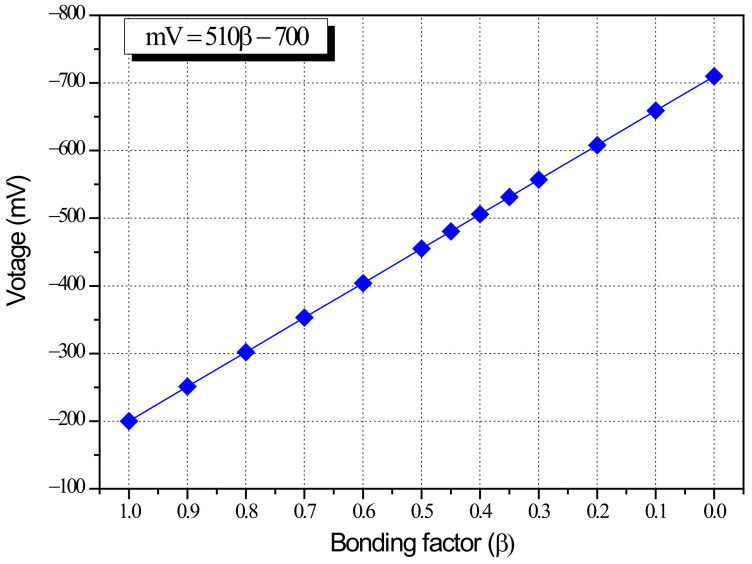
Relationship between bonding factor (β) and shear column specimen average potential difference in terms of voltage (mV).

The bonding factor ϕ in Equations (2) and (10) is a coefficient representing seismic performance reduction in relation to reinforcement corrosion (experiment) and bonding performance (analysis). Since the bonding factor represents the same physical value in the two equations, Equations (2) and (10) can be used to express the average potential difference in voltage (mV) in terms of the bonding factor (β) as shown in Equation (11). Figure 23 is a plot of the β-mV relationship of shear columns.

(11) mV=510β−700

By comparing the relationship between bonding factor (β) and seismic performance reduction factor (ϕ) expressed in Equation (10) and the correlation between average potential difference in voltage (mV) and structural performance reduction factor (ϕ) in Equation (2), the correlation between β and mV in Equation (11) was integrated from the perspective of (β), thus establishing ϕ–β–mV interactions for shear failure-type columns.

## 5. ϕ–β– mV Interactions Based on Structural Experiments and FEA

Using the FEA model for concrete and steel reinforcement described in Section 4, this study determined the effects of weakened bonding caused by reinforcement corrosion on strength-deformability, that is, the correlation between bonding factor (β) and structural performance reduction factor (ϕ) as shown in Figure 22. This section compares the β–ϕ correlation to the mV–ϕ correlation in Figure 14, that is, the relationship between the average potential difference measured by HCP and the seismic performance reduction factor obtained through experiments and proposes mV–β–ϕ interactions of shear columns.

**Figure 24:**
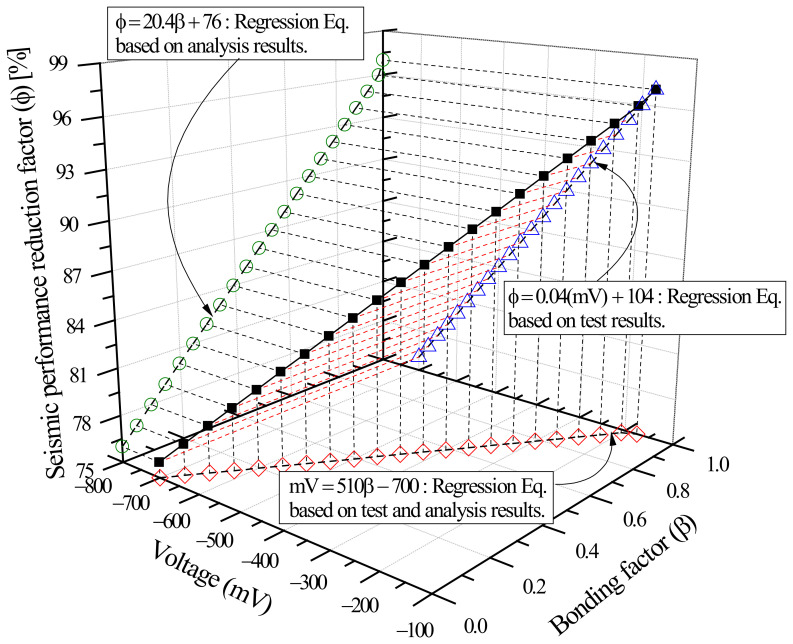
Interaction diagram of performance reduction factor (ϕ), bonding factor (β), and average potential difference in terms of voltage (mV) of corroded columns controlled by shear.

Figure 24 shows the mV–β–ϕ interaction diagram of corrosion-damaged shear columns. Using this diagram, it is possible to evaluate the weakened bonding (β) in relation to extent of corrosion (mV) and the seismic performance reduction factor (ϕ) based on strength-deformability. The results indicate that the proposed method can be utilized for quantitative evaluation of the seismic performance of corrosion-damaged RC members.

## 6. Conclusions

It is extremely complex to estimate the effect of the seismic capacity of corroded structures on the seismic capacity of the entire structural system in terms of force and deformation. This will allow a more accurate assessment of the seismic capacity of RC buildings with corroded beams and columns. Existing techniques for estimating the seismic capacity of RC buildings, however, fail to fully consider the influence of reinforcement corrosion and other performance deterioration of RC structural systems, including beams and columns. The essential purpose of this study is to suggest a realistic technique for estimating the seismic capacity of RC structural systems with corrosion-damaged beams and columns, identifying factors contributing to structural performance deterioration based on strength and deformability for direct, quantitative evaluation of seismic performance.

To achieve the aforementioned objective, the authors examined the effects of reinforcement corrosion on the structural behavior of RC beams and performed experiments demonstrating the correlation between the average potential difference of beam members obtained via HCP and the reduction factor based on strength and deformability [1].

However, current research evaluates the correlation between the extent of corrosion and structural performance deterioration of RC beam members, which are not members that resist lateral force. As such, the results cannot be directly applied to the evaluation of the seismic performance of RC structures containing corrosion-damaged members. To achieve this study’s main purpose of proposing a practical method of evaluating the seismic performance of RC structures comprised of corrosion-damaged members, analytical methods including structural experiments should be applied to corrosion-damaged lateral resisting members, namely, column members of the shear failure type with non-seismic details.

This study performed cyclic loading tests on columns of the shear failure type having reinforcement corrosion to examine the correlation between HCP (mV) before and after corrosion and the seismic performance reduction factor (ϕ). At the same time, FEA was carried out to assess the effects of weakened steel-concrete bonding on seismic performance deterioration based on strength-deformability, and the correlation between bonding factor (β) and seismic performance reduction factor (ϕ) was established. By comparing β–ϕ and mV–ϕ, derived from FEA and structural experiments respectively, this study integrated the correlation of β and mV from the perspective of ϕ, and produced the ϕ–β–mV interaction diagram. The results can be summarized as follows:

(1) Structural experiments of shear failure-type column members in relation to reinforcement corrosion showed that the seismic performance reduction factor (ϕ) defined based on dissipation energy before and after corrosion decreases with a smaller average potential difference (mV) measured by HCP. The reduction factor can be approximated using regression analysis as $\phi =0.04\left(\mathrm{mV}\right)+104$, and the coefficient correlation R^2^ = 0.93 indicated a strong correlation;
(2) The experimental results and FEA results (β = 1) were compared for the control specimen (corrosion-free, SC-C0). The ultimate load in the analysis was 202.9 kN, 105% of the experimental value of 192.4 kN. The ultimate displacement at this point was 12.5 mm in the analysis, about 83% of the experimental value of 15.0 mm. The dissipation energy was found to be 2105.9 kN-m, which is highly similar to the experimental value of 2106.7 kN. The analysis results using VecTor2 were consistent with experimental observations. The proposed FEA model can be seen as effective in examining the effects of weakened bonding on structural performance reduction based on strength-deformability, that is, the correlation between bonding factor (β) and seismic performance reduction factor (ϕ);
(3) The bonding factor (β) and structural performance reduction factor (ϕ) can be approximated using ϕ=20.4β+76, obtained by regression analysis. The correlation coefficient of β-ϕ was R^2^=0.93, which indicates a very strong correlation. In addition, the β–ϕ correlation and the correlation between potential difference (mV) and structural performance reduction factor (ϕ), that is, $\phi =0.04\left(\mathrm{mV}\right)+104$, were compared in order to express the β–mV correlation in terms of the seismic performance reduction factor (ϕ) as mV=510β−700;
(4) The β–ϕ correlation of each column specimen based on FEA was compared to the mV–ϕ correlation, which is the relationship between average potential difference, quantitatively measured by HCP, and seismic performance reduction factor, derived from structural experiments. The mV–β–ϕ interaction diagram of shear failure-type columns was thus established. Using the mV–β–ϕ interaction diagram, it is possible to evaluate the weakened bonding (β) in relation to the extent of corrosion (mV) and seismic performance reduction factor (ϕ) based on strength-deformability. The results indicate that the proposed method can be utilized for quantitative evaluation of the seismic performance of corrosion-damaged RC members;
(5) To develop practical, commercial methods for the evaluation of the seismic performance of corrosion-damaged RC structures, it is necessary to develop techniques for quantitative measurements based on HCP, estimation of seismic performance reduction factor, and nonlinear analysis of the seismic performance of the entire structure in consideration of performance deterioration. As a recommendation for future work, it is recommended to apply multiple NDE (Nondestructive Evaluation) techniques rather than HCP alone to increase the reliability of the interaction diagram proposed in this study.

## Figures and Tables

**Table 1:** Overview of the major previous research related to this study.

Reference	Member Type	Methodology	Corrosion Levels (%)	Residual Capacity Range (%)
J. S. Jung et al., (2019) [1]	Beam	Test	Not provided	70.0–100.0
D. M. Frangopol et al., (1997) [4]	Girder	Reliability-based design	Not provided	80.0–100.0
A. Shamsad (2003) [5]	-	Non-destructive test	Not provided	Not provided
H.S. Lee et al., (2009) [7]	Cube (Pull-out)	Test	Not provided	85.0–100.0
A. Kaveh et al., (2019) [10]	Reinforcement	Tensile test and Analysis	0.0–24.5	Not provided
J. G. Cabrera (1996) [16]	Cube (Pull-out) and Beam	Test	0.0–12.6 and 0.0–7.8	23.8–100.0 and 71.8–100.0
R. Capozucca (1995) [17]	Beam and Column	Analysis	-	-
Bhargava, K et al., (2008) [19]	Beam	Test	1.3–10.0	18.9–100.0
Yang, X et al., (2012) [22]	Beam	FEA	0.0–11.0	30.0–100.0
Ballim, Y et al., (2003) [23]	Beam	Test	0.0–8.47	55.0–100.0
Torres-Acostaa, A et al., (2007) [24]	Beam	Test	0.0–16.1	27.2–100.0

**Table 2:** Loading cycles.

Loading Step	1	2	3	4	5	6
Loading cycles	1~3	4~6	7~9	10~12	13~15	16~18
Drift angle (R) (%)	0.1	0.2	0.3	0.4	0.5	0.67
Lateral drift (mm)	1.5	3.0	4.5	6.0	7.5	10
**Loading step**	**7**	**8**	**9**	**10**	**11**	**12**
Loading cycles	19~21	22~24	25~27	28~30	31~33	34~36
Drift angle (R) (%)	0.83	1	1.17	1.33	1.67	2
Lateral drift (mm)	12.5	15	17.5	20	25	30

**Table 3:** Average potential difference in voltage.

Column Specimens	Average Potential Difference in Voltage mV CSE (Copper Sulfate Electrode)
SC-C0	−125
SC-C1	−405
SC-C2	−545
SC-C3	−575
SC-C4	−605

Note: SC indicates columns controlled by shear and C shows corrosion-damaged RC columns.

**Table 4:** Test results for each specimen.

Beam	Average Potential Difference in Voltage	Ultimate		Dissipation Energy (kN-mm)	Seismic Performance Reduction Factor ϕ (%)
	mV CSE (Copper Sulfate Electrode)	Load (kN)	Displacement (mm)		
SC-C0	−125	192.4	15.0	2106.7	100.0
SC-C1	−405	204.4	12.5	1861.8	88.4
SC-C2	−545	192.3	15.0	1843.8	87.5
SC-C3	−575	190.7	12.5	1778.1	84.4
SC-C4	−605	190.3	12.5	1718.9	81.5

**Table 5:** Analytical models used in FEA modeling.

Material	Material Property	Model
Concrete models	Compression pre-peak	Hognestad (parabola)
	Compression post-peak	Kent/Park
	Compression softening	Vecchio 1992-A (e1/e2-form)
	Tension stiffening	Lee 2011 (w/post yield)
	Tension softening	Not considered
	FRC tension	Not considered
	Confined strength	Kupfer/Richart
	Dilation	Variable–isotropic
	Cracking criterion	Mohr–Coulomb (Stress)
	Crack stress calculation	Basic (DSFM/MCFT)
	Crack width check	Stability check omitted
	Crack slip calculation	Vecchio-Lai
	Hysteretic response	Nonlinear w/plastic offsets
Reinforcement models	Hysteretic response	Elastic-hardening (curvilinear)
	Dowel action	Tassios model (crack slip)
	Buckling	Buckling (Akkaya 2012 (modified Dhakal-Maekawa))
Bond models	Concrete bond	Eligehausen

**Table 6:** Analysis results for each case of shear column.

Cases	Bonding Factor (β)	Ultimate		Dissipation Energy (kN-mm)	Seismic Performance Reduction Factor ϕ (%)
		Load (kN)	Deflection (mm)		
Case-1	1.00	202.9	12.5	2105.9	100.0
Case-2	0.9	201.5	12.5	1958.5	93.0
Case-3	0.8	199.8	12.5	1912.8	90.8
Case-4	0.7	199.1	12.5	1879.3	89.2
Case-5	0.6	198.5	12.5	1865.2	88.6
Case-6	0.5	198.1	12.5	1840.4	87.4
Case-7	0.45	201.1	12.5	1810.1	86.0
Case-8	0.4	199.7	12.5	1760.9	83.6
Case-9	0.35	196.7	12.5	1713.5	81.4
Case-10	0.3	195.4	12.5	1695.1	80.5
Case-11	0.2	195.1	12.5	1690.6	80.3
Case-12	0.1	194.3	12.5	1654.5	78.6
Case-13	0.0	193.4	12.5	1637.7	77.8

## Data Availability

All datasets generated during this study are available from the corresponding author on reasonable request.
